# Establishment of LC-MS/MS Method for Determination of GMDTC in Rat Plasma and Its Application in Preclinical Pharmacokinetics

**DOI:** 10.3390/molecules28031191

**Published:** 2023-01-25

**Authors:** Wei Hu, Zhi-Yong Zhong, Yu-Ting Gao, Xue-Feng Ren, Hai-Yang Liu, Xiao-Jiang Tang

**Affiliations:** 1 Key Laboratory of Functional Molecular Engineering of Guangdong Province, School of Chemistry and Chemical Engineering, South China University of Technology, Guangzhou 510641, China; 2 Jianersheng (Zhuhai) Pharmtech Co., Ltd., Zhuhai 519040, China; 3 Guangdong e-fang Pharmaceutical Co., Ltd., Foshan 528244, China; 4 Southern Medical University, Guangzhou, Guangzhou 510515, China

**Keywords:** GMDTC, cadmium, pharmacokinetic, LC–MS/MS, bioanalytical method

## Abstract

Sodium (S)-2-(dithiocarboxylato((2S,3R,4R,5R)-2,3,4,5,6-pentahydroxyhexyl)amino)-4(methylthio)butanoate (GMDTC) is the first compound to use cadmium repellent as an indication. In this paper, we established and validated a bioanalytical method for the determination of GMDTC in rat plasma, and used it to determine the drug concentrations in the plasma of rats after intravenous dosing in different genders and dosages. After pretreating the plasma samples with an acetonitrile–water–ammonia solution (70:30:1.25, *v*/*v*/*v*), liquid chromatographic separations were efficiently achieved with a XBridge C18 column using a 5 min gradient system of aqueous ammonium bicarbonate and 95% acetonitrile–water solution (95:5, *v*/*v*) as the eluent. The GMDTC and metolazone (internal standard, IS) detection were carried out using high-performance liquid chromatography coupled with triple quadrupole mass spectrometry (LC–MS/MS), monitored at *m*/*z* 390.06–324.1 (for the GMDTC, tR: 2.03 min) and *m*/*z* 366.0–259.2 (for IS, tR: 3.88 min). The GMDTC was stable under various testing conditions, and this analytical method conforms to the verification standard of biological analysis methods. The half-life (t_1/2_) was determined to be 0.54–0.65 h for the intravenous, mean distribution volume and clearances were 1.08–2.08 L/kg and 1–3 L/h/kg, respectively. The AUC_0-t_ and AUC_0-∞_ found after increasing the dosage exhibited a linear relationship with the administered dose. There were no statistically significant differences in the values obtained for the different genders at dosages of 50, 100 and 250 mg/kg, respectively (*p* > 0.05). This is the first report of a bioanalytical method to quantify GMDTC in rat plasma using LC–MS/MS, which provides useful information for the study of its pharmacological effects and clinical applications.

## 1. Introduction

Cadmium (Cd) is an environmental pollutant that threatens human health. It occurs naturally and is widely distributed throughout the world [1]. It can be released into the environment through anthropogenic activities, which results in the accumulation of cadmium in the air, water and soil year by year [2]. Long-term exposure to cadmium in humans can have toxic effects in different organ systems, including urinary, reproductive, cardiovascular, skeletal and respiratory system toxicity [3]. It can lead to kidney disease [4,5,6], diabetes [7], impaired reproductive function [8], cardiovascular diseases [9], osteoporosis [10,11] and an increased risk of cancer [12,13]. Cadmium has long been listed as the sixth most dangerous substance to human health by the American Toxic Substances and Disease Registry (ATSDR) [14], and the International Agency for Research on Cancer (IARC) classified cadmium as a known human carcinogen (Group I) as early as 1993 [15,16]. The World Health Organization (WHO) also declared that cadmium exposure is a major global public health problem, and revealed the main sources and harms of cadmium exposure [17]. Therefore, the prevention and treatment of cadmium pollution and poisoning are serious problems.

The kidney is the organ most affected by cadmium toxicity. Even at low levels, long-term cadmium exposure may cause renal dysfunction. So far, there is no specific antidote for cadmium poisoning [18], and the existing drugs such as EDTA and other complexing agents are not effective in removing cadmium accumulated in the kidney.

**Figure 1:**
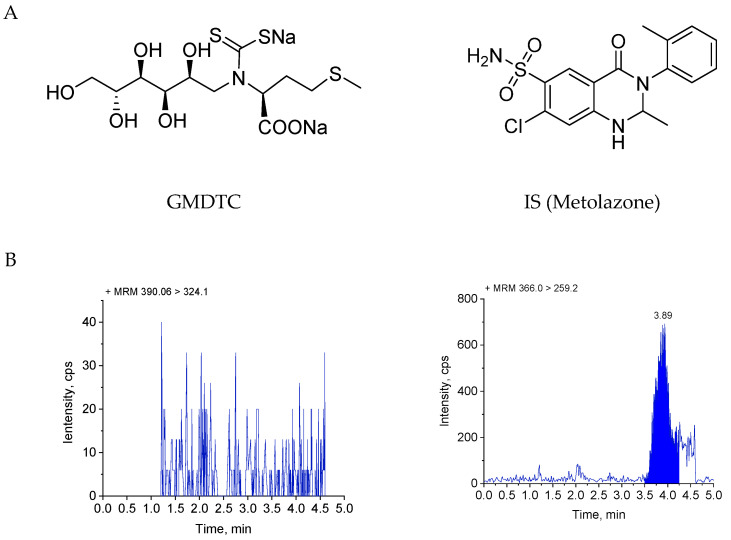

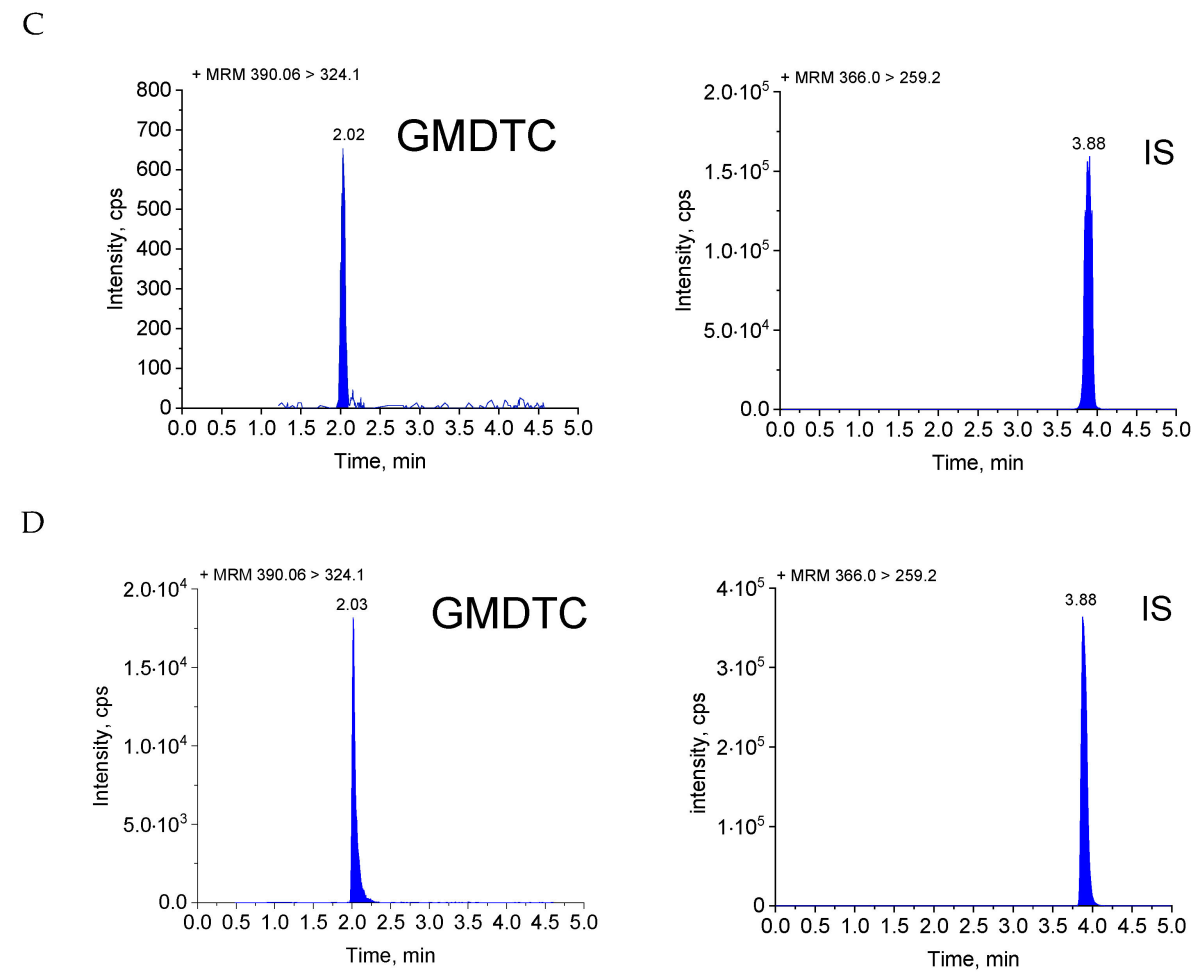
Chemical structures and typical MRM chromatograms of GMDTC (left, *m*/*z* 390.06→324.1) and IS (right, *m*/*z* 366.0→259.2). (**A**) Chemical structures of GMDTC and metolazone, (**B**) blank rat plasma, (**C**) blank rat plasma spiked with GMDTC (LLOQ, 50 ng/mL) and IS (at the level of use, 50 ng/mL), (**D**) plasma sample taken 0.133 h after injection administration of 50 mg/kg GMDTC to the rats.

GMDTC (Figure 1A) is a new chelating agent that uses an innovative sodium–glucose cotransporter 2 (SGLT2) and glucose transporter type 2 (GLUT2) coupled transport mechanism to expel cadmium from the kidney [19]. After intravenous infusion into the blood, the GMDTC is filtered out through the glomerulus into the renal tubules, reabsorbed through the SGLT2 into the renal tubular cells, combined with cadmium ions to form Cd–GMDTC complex, and then enters the blood through the GLUT2. It is then filtered out through the glomerulus and excreted through the urine. The renal cadmium elimination rate was as high as 94% in 4 weeks. Also, the toxicity of GMDTC is low, and no significant toxic side effects were found in acute toxicity and long-term toxicity experiments in various animal models [19]. GMDTC is a good cadmium repellent, and its clinical trials application in China is under technical evaluation (No.: CXHL2200667, China).

During the development of new drugs, pharmacokinetic studies are very important for determining dosing and dosing intervals, and they help us to better understand the mechanism of action of the drug and provide very useful information for clinical applications. In this study, we established and verified a rapid, stable and effective LC–MS/MS method to determine GMDTC concentrations in rat plasma, and studied the pharmacokinetics of three different dosages in male and female rats by tail vein intravenous administration. This is the first report of a bioanalytical method for the quantification of GMDTC in rat plasma by LC–MS/MS.

## 2. Results

### 2.1. Method Validation

The LC–MS/MS method validation results conform to the U.S. Food and Drug Administration (FDA, Silver Spring, MA, USA) bioanalytical method validation guidelines [20].

Figure 1 illustrates the MRM chromatograms of the blank plasma samples spiked with GMDTC and metolazone (IS), as well as their chemical structures, and rat plasma obtained from the pharmacokinetic study (0.133 h after intravenous administration of GMDTC at a dosage of 50 mg/kg). There were no obvious impurities or endogenous substances interfering with the detection of the GMDTC in each blank sample to be tested, and the interference with the IS was less than 5%. There was no interference in the GMDTC retention time after the blank sample to which the upper limit of quantification (ULOQ) IS was added; after the blank sample to which the upper limit of quantification (ULOQ) GMDTC was added, the interference peak area at the retention time of the IS was less than 5% of the average area of the standard curve and the internal standard of the quality control (QC) sample. The retention times (tR) for the GMDTC and IS were 2.03 min and 3.88 min.

The calibration curve for the GMDTC in the rat plasma showed a linear relationship in the range of 50–5000 ng/mL. The correlation coefficients (r) were not less than 0.99 with a representative linear regression equation of y = 3.37e−5x + 3.52e−5 (r = 0.9995) for the GMDTC. The LLOQ of this method was 50 ng/mL.

Table 1 shows the intra-day and inter-day precision and accuracy of the evaluation with the LLOQ and three QC samples on the same day and different days. The precision and accuracy of the intra-day and inter-day were 1.64% to 7.47% and 2.40% to 7.87%, respectively. The results satisfied all the criteria and proved the reliability of the developed method.

The recovery rate and matrix effect of GMDTC using this method are shown in Table 2. The relative recovery rate of the samples ranged from 104.37% to 108.30%, and the RSD was less than 15% (2.84~4.13%). The RSD of the matrix factor normalized by IS was less than 4.15%. The results show that the analytical method meets the requirements.

After each sample is tested, the system is washed with a 50% aqueous methanol solution for three minutes to eliminate carry-over. In this dilution reliability verification test, in addition to the verification of the dilution of 10–100 times, we also conducted the verification of the dilution test with a concentration close to the LQC (150 ng/mL), using a nominal concentration of 80,000 ng/mL and dilution of 500 times. Table 3 shows when the nominal concentration ranges from 6000–80,000 ng/mL, the 10–500 dilution times will not affect the testing accuracy and precision. The accuracy of the concentration measurement was 4.03~12.40%, and the RSD was ≤ 3.02%. According to the FDA’s acceptance standard, the accuracy and precision at each level are within 15%, which indicates that reanalysis of the samples with a higher concentration than the ULOQ can be completed by proper dilution.

At the concentrations of 150 and 4000 ng/mL, the stability of the GMDTC in rat plasma was tested under different conditions. This information is shown in Table 4. According to the peak ratios of all the samples, the stability sample of the stored solution was compared with the newly prepared standard solution. A concentration range of about 15% in the new standard solution was considered as acceptable stability. The RSD was 0.76~5.56%, and the RE was −9.28~11.98%. The plasma samples were stable under light-proof conditions for 7 h at 2–8 °C, 28 days at −60 °C and after three freeze–thaw cycles (−60 °C to 2–8 °C). The content of GMDTC in the extracts was also stable when placed in an autosampler at 4 °C for 92 h.

### 2.2. Pharmacokinetic Application

**Figure 2:**
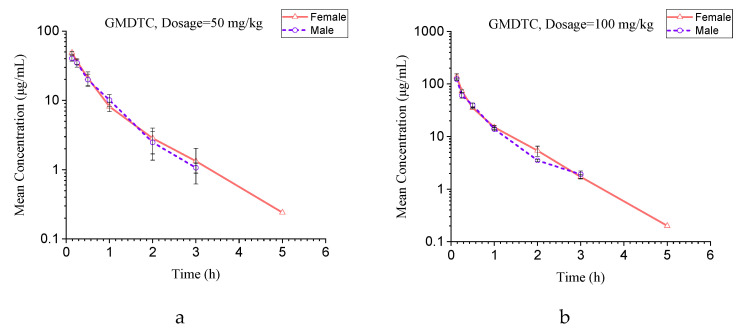

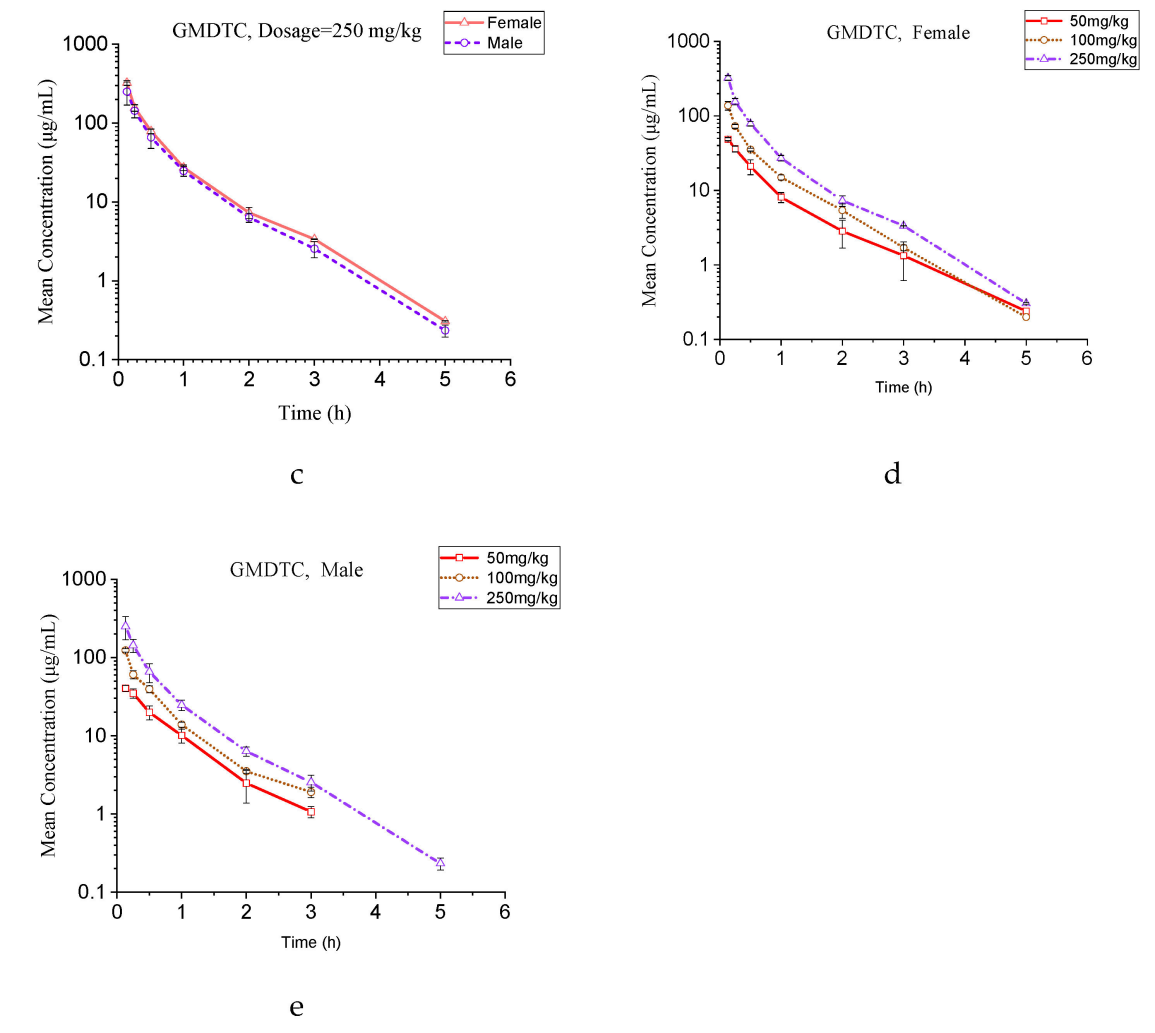
(**a**) Concentration–time curve of GMDTC in plasma of male and female rats at 50 mg/kg. (**b**) Concentration–time curve of GMDTC in plasma of male and female rats at 100 mg/kg. (**c**) Concentration–time curve of GMDTC in plasma of male and female rats at 250 mg/kg. (**d**) Concentration–time curve of GMDTC in plasma of female rats at dosages of 50, 100 and 250 mg/kg. (**e**) Concentration–time curve of GMDTC in plasma of male rats at dosages of 50, 100 and 250 mg/kg.

Pharmacokinetic studies of GMDTC were performed to demonstrate the applicability of the established LC–MS/MS method. In this study, adult male and female SD rats were given intravenous dosages of GMDTC at 50, 100 and 250 mg/kg. For the plasma samples in the high dosage group whose concentration was above the curve range, we diluted blank plasma by 10 to 500 times to reach the concentration in the linear range, and then analyzed it according to the established method. After a single intravenous infusion of each dose group (50, 100 and 250 mg/kg), the concentration–time curves of the GMDTC in the plasma of each rat is shown in Figure S1, and the plasma concentration of the GMDTC at each time point is shown in Tables S1–S3. The plasma concentration–time curves of the GMDTC are shown in Figure 2. Following intravenous injection, the GMDTC showed a typical nonlinear response pattern with an increasing slope as its dosages (50, 100 and 250 mg/kg, respectively) increased.

The main GMDTC pharmacokinetic parameters obtained are summarized in Table 5, and the pharmacokinetic parameters of each rat are shown in Tables S4–S6.

**Figure 3:**
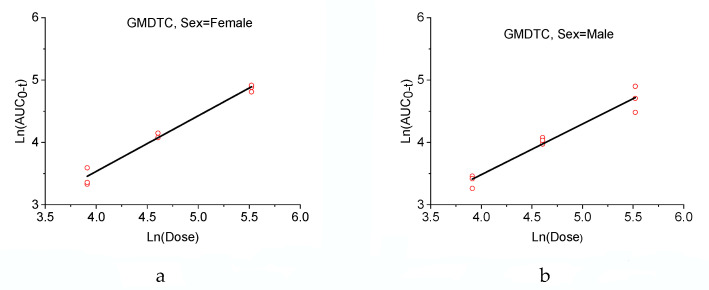
(**a**) Female rats plasma Ln(AUC_0-t_)–Ln(Dose) regression curve of GMDTC at dosages of 50, 100 and 250 mg/kg; (**b**) Male rats plasma Ln(AUC_0-t_)–Ln(Dose) regression curve of GMDTC at dosages of 50, 100 and 250 mg/kg.

The Ln(AUC_0-t_) were linear with the Ln(Dose) (male and female, respectively), as shown in Figure 3. The equation for the females was Ln(AUC_0-t_) = 0.8924 Ln(Dose) − 0.0346, with correlation coefficients r^2^ = 0.9803, and the equation for male was Ln(AUC_0-t_) = 0.8131 Ln(Dose) − 0.2300, with correlation coefficients r^2^ = 0.9532. It shows that there is a linear relationship between the dosage and blood concentration.

## 3. Discussion

In this study, we successfully applied an effective LC–MS/MS method to study the pharmacokinetics of GMDTC in male and female rats. This is the first report on the pharmacokinetics of GMDTC in animals.

We conducted several experiments to develop and optimize the LCMS to quantify the GMDTC in rat plasma. As a compound with good water solubility and high polarity, the GMDTC was unstable in the column and could not be detected stably when a conventional C18 column was used. After several attempts, we chose the XBridge C18 column, with better water resistance and alkaline resistance. The GMDTC was easily decomposed in methanol and stable in aqueous acetonitrile, so we used an acetonitrile–water solution (95:5, *v*/*v*) as the eluent for the organic phase. GMDTC is a sodium salt compound with a very short retention time on the reversed-phase column, which does not meet the analytical requirements. We added 10 mmol of sodium bicarbonate to the aqueous phase to increase its retention time to meet our analytical needs. GMDTC is degraded in plasma. In the study of drug stability, we found that GMDTC is stable in ammonia water. After continuous experiments, when whole blood was treated with acetonitrile–water–ammonia in the volume ratio of 70:30:1.25 and the Na_2_EDTA anticoagulant was used, a stable recovery rate of GMDTC was obtained. Therefore, the blood samples in the preparation of blank plasma and the pharmacokinetics of rats were treated by this method. The previous literature suggests that GMDTC is unstable [21]. We found that the purity of the GMDTC solution decreased from 96.5% to 80.0% after 12 h of light. The mass spectrometric response of 50 ug/mL GMDTC in plasma at room temperature decreased by 58% at 1 h and 74% at 3 h. The whole process needs to be performed at low temperature and under light protection.

In our pharmacokinetic study, GMDTC was administered by tail vein injection to male and female rats at doses of 50, 100 and 250 mg/kg, respectively, and blood was collected from the jugular vein to quantify the GMDTC in the blood using established methods. The main GMDTC pharmacokinetic parameters, such as C_max_, AUC_0-t_ and AUC_0-∞_, increased with the increasing dosage and had a linear relationship; there were no statistically significant (*p* > 0.05) differences in the values obtained between the male and female rats. The other pharmacokinetic parameters were T_max_ (0.133 h), t_1/2_ (0.54–0.65 h), Cl (1–3 L/h/kg), mean V_d_ (1.31–2.08 L/kg), mean V_ss_ (0.99–1.32 L/kg), mean MRT_0-t_ (0.51–0.65 h) and mean MRT_0-∞_ (0.54–0.72 h). The T_max_ was 0.133 h, and the C_max_ of the GMDTC reached its maximum value after the administration. There were no statistically significant differences in the t_1/2_ values obtained for the various dosages, which is consistent with the first-order kinetic characteristics. Compared to the liver blood flow of 3.3 L/h/kg [22], the clearance rate of the GMDTC in the rats was moderate. The values of V_d_ and V_ss_ showed that the GMDTC was evenly distributed in the rats. The half-life of this drug in rats is only about 30 min, which also explains the pharmacodynamic study of Tang and colleagues [19]: the expelling effect of GMDTC on cadmium was 6 h after administration, and 7–24 h hardly increased cadmium excretion. There were no statistically significant differences in the T_max_, t_1/2_, Cl, V_d_, V_ss_, MRT_0-t_ and MRT_0-∞_ values obtained for the various dosages and genders, which indicates that the elimination of GMDTC was unsaturated, and therefore the administration concentration of GMDTC can be increased.

This study provides a reference for further research on pharmacokinetics, tissue distribution, metabolite analysis and excretion of GMDTC between different species, and provides useful information for further research on its pharmacological actions and clinical application.

## 4. Materials and Methods

### 4.1. Chemicals and Reagents

The GMDTC (purity > 95% by HPLC-UV, content > 80% by NMR) was synthesized at Asymchem Laboratories (Tianjin) Co., Ltd. (Tianjin, China). The metolazone (99.88%), used as the internal standard (IS), was purchased from Dalian Meilun Biotech Co., Ltd. (Dalian, China). The methanol and acetonitrile for the gradient HPLC was purchased from Thermo Fisher Scientific Inc. (Tustin, CA, USA). Ammonium bicarbonate, ammonia, and disodium dihydrogen ethylenediaminetetraacetate were purchased from Sinopharm Chemical Reagent Co., Ltd. (Shanghai, China.).

### 4.2. Animals

All the Sprague–Dawley (SD) rats (220 ± 20 g) used for the experiments were obtained from Zhejiang Vital River Laboratory Animal Technology Co., Ltd. (Hangzhou, China). Eighteen SD rats (nine male, body weight 220–240 g; nine female, body weight 200–220 g) were raised in an environment free of specific pathogens, with 12 h light–dark cycles. They were fed and watered freely. All the animal experiments were performed in accordance with the Guide for the Care and Use of Laboratory Animals.

### 4.3. Analytical System and Chromatographic Conditions

A QTRAP 5500 LC–MS/MS system (AB Sciex, Framingham, MA, USA) equipped with an electrospray ionization interface was used to quantify the IS and GMDTC. The substances were separated on a reversed-phase column (XBridge C18, 4.6 × 150 mm internal diameter, 5μm particle size; Waters, Ireland) at 35 °C. The mobile phase consisted of a 5 min gradient system combining 10 mmol/L aqueous ammonium bicarbonate (A) and 95% aqueous acetonitrile (B), at a flow rate of 0.6 mL/min, as follows: 0–0.2 min, 40% B; 0.2–2.1 min, 40–90% B; 2.1–4 min, 90% B; 4–4.1 min, 90–40% B; 4.1–5 min, 40% B. Subsequently, the column was re-equilibrated with 40% B for three minutes. The injection volume was 5 μL, and the samples were conserved at 4 °C in the auto-sampler. The effluent of 0–1.2 min and 4.6–5 min from the column did not enter the ion source.

The mass spectrometer used multiple reaction monitoring (MRM) (positive-ion mode). The mass spectrometry parameters, including the declustering potential (DP), collision energy (CE), collision cell exit potential (CXP) and Q1 and Q3 voltages were optimized to obtain the highest sensitivity for the monitored transitions, declustering potential (DP), 90 V for the GMDTC and 75 V for the IS; collision energy (CE), 25 V for the GMDTC and 30 V for the IS. The mass transition occurred at *m*/*z* 390.06–324.1 for the GMDTC and 366.0–259.2 for the IS. The final optimized parameters are summarized in Table 6. The other parameters were as follows: ion spray voltage, 5500 V; source temperature, 600 °C; ion source gas I at 55 psi, gas II at 55 psi, curtain gas at 40 psi. The chromatograms were integrated, and the data were acquired by the Analyst 1.7.1 software (AB SCIEX, Framingham, MA, USA).

### 4.4. Plasma Sample Preparation and Calibration Curves

An appropriate volume of blank rat whole blood (Na_2_EDTA anticoagulation) was added to an appropriate volume of acetonitrile–water–ammonia solution (70:30:1.25, *v*/*v*/*v*), in which the volume of solvent is three-times that of whole blood, mixed and centrifuged at approximately 16,000 g for 10 min at 2–8 °C, and the supernatant was aspirated and used as a blank biological matrix.

The standard solution of GMDTC was prepared individually in 0.1% ammonia–water solution (1 mg/mL). The GMDTC standard solution was serially diluted in acetonitrile –water–ammonia solution (70:30:1.25, *v*/*v*/*v*) to obtain a concentration range from 1 ug/mL to 100 ug/mL. The working solution of GMDTC was added into the blank matrix to prepare the standard solutions of GMDTC with concentrations of 50, 100, 200, 500, 1000, 1250, 2500 and 5000 ng/mL. Quality control samples LLOQ, LQC, MQC and HQC (50, 150, 800 and 4000 ng/mL) were prepared by the same method.

The standard solution of metolazone (IS) was prepared individually in acetonitrile (1 mg/mL). The IS standard solution was diluted in a methanol–acetonitrile solution (80:20, *v*/*v*) to obtain a concentration of 50 ng/mL.

The plasma samples were thawed at 4 °C in the dark before analysis. A volume of 100 μL of working IS solution was added to the 100 μL plasma in 1 mL disposable polypropylene microcentrifuge tubes, and mixed and centrifuged at approximately 3200 g for 15 min at 2–8 °C. Next, 200 μL Na_2_EDTA-ammonia–water solution (0.336:1.25:100, *w*/*v*/*v*) was added to 50 μL supernatant and vortex-mixed. The supernatant (5 μL) was injected into the LC–MS/MS system.

### 4.5. Method Validation

The LC–MS/MS method was validated according to the U.S. Food and Drug Administration’s (FDA) Bioanalytical Method Validation Guide [20], and included the determination of selectivity, linearity, lower limit of quantitation (LLOQ), accuracy, precision, matrix effect, recovery, stability, carryover effect and dilution integrity.

#### 4.5.1. Selectivity

In order to exclude the interference of endogenous substances, the chromatograms of six different batches of blank rat plasma, blank plasma spiked with GMDTC and IS, and plasma samples obtained after the use of GMDTC were used to evaluate the selectivity.

#### 4.5.2. Linearity and Lower Limit of Quantification (LLOQ)

Calibration curves must contain a blank sample, a zero sample and six concentration points tested for at least 3 days. The linearity of the method was evaluated by plotting the peak area ratio of analytes to the IS versus the nominal concentration of analytes. The LLOQ in a calibration curve could be quantified with precision within 20%, and the other samples no more than 15%.

#### 4.5.3. Precision and Accuracy

Relative standard deviation (RSD) was used to express precision, and relative error (RE) was used to express accuracy. Intraday precision and accuracy are determined from the standard curve and QC samples of four concentrations (LLOQ, LQC, MQC and HQC) on the same day; inter-day precision and accuracy are determined from QC samples of four concentrations for at least 3 days, using six samples for each concentration level. The intraday and inter-day precision and accuracy should be determined at no more than 15%, and the LLOQ precision and accuracy should be determined at no more than 20%.

#### 4.5.4. Extraction Recovery and Matrix Effect

The extraction recovery was determined by comparing the peak areas of QC samples at three concentrations with those of samples spiked with GMDTC and IS to equivalent concentrations in a blank matrix after extraction.

The matrix effect was determined by comparing the peak area of the GMDTC and IS spiked in post-extracted blank plasma with those of neat samples at the same concentration. The RSD of the matrix factor, normalized by the IS calculated from the six batches of matrix, was measured at the LQC and HQC levels. The RSD of the recovery and matrix effect should not be higher than 15%.

#### 4.5.5. Carryover Effect

After running the ULOQ sample (5000 ng/mL), a blank sample (treated matrix, without the addition of GMDTC and IS) was run to examine the carryover effect. The interference peak area at the retention time should not exceed 20% of the LLOQ and 5% of the IS.

#### 4.5.6. Dilution Integrity

The plasma samples with GMDTC exceeding the ULOQ (5000 ng/mL) were diluted 10, 100 and 500 times with blank plasma. The obtained concentrations were compared with the nominal concentration to check if the dilution affects the accuracy and precision or not. The RE and RSD should be within 15%.

#### 4.5.7. Stability

The stability of the analytes was evaluated by QC samples at two concentrations (LQC and HQC). Short-term stability was evaluated by placing the treated samples in the dark at 2–8 °C. Long-term stability was determined by keeping the samples below −60 °C in the dark. The autosampler stability was investigated by placing the samples in the autosampler room. The stability of the freeze–thaw cycle was analyzed after three cycles of freezing the samples for no less than 12 h each.

### 4.6. Application to Pharmacokinetic Study

In the published literature on the efficacy of GMDTC [19], the dosage in rabbit is 108, 216 and 433 mg/kg. We gave the lowest possible dose in the first pharmacokinetic experiment, and the doses in the rats were 50, 100 and 250 mg/kg. Considering that GMDTC is an alkaline injection, and the pH of the 10 mg/mL GMDTC solution was greater than 8.3, the rats were administered intravenous infusions in the tail veins with a maximum dose of 20 mL/kg, and the time was about 8 min. Eighteen SD rats (nine female and nine male) were raised in an environment free of specific pathogens. These rats were randomly divided into three groups (*n* = 6 per group, 3 female and 3 male). After intravenous infusion of a single dosage (50, 100 and 250 mg/kg, respectively) of GMDTC, approximately 0.25 mL blood samples were collected from the jugular vein at 0.133 (blood was collected immediately after administration), 0.25, 0.5, 1, 2, 3, 5, 8 and 12 h.

Prior to the blood collection, brown centrifuge tubes containing 20 µL Na_2_EDTA (150 mg/mL) were cooled to 2–8 °C in a refrigerator or ice box filled with crushed ice. The collected blood was added to a brown tube and manually inverted at least 5 times. A quantity of 200 µL of whole blood was transferred into a brown tube containing 600 µL of acetonitrile–water–ammonia solution (70:30:1.25, *v*/*v*/*v*) using a precision pipette. The samples were mixed and centrifuged at approximately 16,000 g for 10 min at 2–8 °C, and then stored below −60 °C in the dark until analysis. All the samples were placed in an ice box filled with crushed ice during transportation and were managed using the Watson LIMS 7.5 system

The pharmacokinetic parameters were determined by analyzing the plasma concentration–time profiles and were calculated using WinNonlin (Version10.0, Mountain View, CA, USA) according to a noncompartmental model. The values are expressed as the mean ± SD.

## 5. Conclusions

This is the first report on the bioanalytical method for the determination of GMDTC in rat plasma using LC–MS/MS, and the GMDTC remained stable throughout the analysis with an LLOQ of 50 ng/mL. We used this method for a preliminary preclinical study to determine GMDTC concentrations in the plasma of male and female rats at three different dosages (50, 100 and 250 mg/kg, respectively) of intravenous GMDTC and provided pharmacokinetic parameters under these conditions.

## Figures and Tables

**Table 1:** Precision and accuracy values of GMDTC in rat plasma.

Nominal Concentration (ng/mL)	Measured Concentration (ng/mL)	Precision (RSD, %)	Accuracy (RE, %)
Intra-day batch (*n* = 6)			
50	51.20 ± 3.82	7.47	2.40
150	160.80 ± 8.87	5.52	7.20
800	859.09 ± 13.61	1.64	3.64
4000	4196.08 ± 123.90	2.95	4.90
Inter-day batch (*n* = 36)			
50	53.93 ± 3.61	6.70	7.87
150	159.53 ± 11.43	7.17	6.36
800	855 ± 54.56	6.38	6.88
4000	4245.33 ± 238.89	5.63	6.13

**Table 2:** Recovery and matrix effect values of GMDTC in rat plasma.

Concentration (ng/mL)	Extraction Recovery		Matrix Effect	
	Mean (%)	RSD (%)	Mean (%)	RSD (%)
150	105.86	3.17	1.04	4.15
800	108.30	4.13		
4000	104.37	2.84	0.93	2.93

**Table 3:** The dilution reliability of 10, 100 and 500-fold dilution of plasma samples.

Dilution Times	Nominal Concentration (ng/mL)	Measured Concentration (ng/mL)	Precision (RSD, %)	Accuracy (RE, %)
10	6000	6753.99 ± 137.86	2.04	12.40
100	60,000	66,991.62 ± 1070.94	1.60	11.65
500	80,000	83,227.13 ± 2514.19	3.02	4.03

**Table 4:** The stability of GMDTC under various storage conditions.

Stability Studies	Nominal Concentration (ng/mL)	Measured Concentration (ng/mL)	Precision (RSD, %)	Accuracy (RE, %)
Short-term stability ^a^	150	156.03 ± 1.18	0.76	4.02
	4000	4042.76 ± 81.92	2.02	1.07
Long-term stability ^b^	150	154.41 ± 8.58	5.56	2.94
	4000	3958.67± 73.22	1.85	−1.03
Freeze–thaw stability ^c^	150	167.98 ± 1.67	1.00	11.98
	4000	4476.71 ± 122.19	2.73	11.92
Post-preparative stability ^d^	150	137.78 ± 6.61	4.80	−8.14
	4000	3628.76 ± 69.53	1.92	−9.28

^a^ Samples were stored at 2–8 °C and avoided light for 7 h to test short-term stability. ^b^ Samples were stored at −60 °C and avoided light for testing long-term stability. ^c^ Samples after three freeze–thaw cycles form −60 °C to 2–8 °C for testing freeze–thaw stability. ^d^ Samples in auto-sampler at 4 °C for testing post-preparative stability.

**Table 5:** Main pharmacokinetic parameters of GMDTC.

Parameters	Units	50 mg/kg		100 mg/kg		250 mg/kg	
		Male	Female	Male	Female	Male	Female
t_1/2_	(h)	0.55 ± 0.04	0.65 ± 0.06	0.54 ± 0.03	0.65 ± 0.04	0.62 ± 0.01	0.64 ± 0.02
T_max_	(h)	0.133 ± 0.00	0.133 ± 0.00	0.133 ± 0.00	0.133 ± 0.00	0.133 ± 0.00	0.133 ± 0.00
C_max_	(μg/mL)	40.40 ± 3.72	48.78 ± 2.30	123.32± 6.25	138.05 ± 17.98	250.77 ± 81.50	324.36 ± 22.68
AUC_0-t_	(h·μg/mL)	29.48 ± 2.97	31.01 ± 4.63	56.20 ± 2.88	61.95 ± 2.49	111.14 ± 22.90	130.53 ± 7.05
AUC_0-∞_	(h·μg/mL)	30.19 ± 3.18	31.60 ± 4.35	57.31 ± 2.88	63.12 ± 3.33	111.36 ± 22.86	131.69 ± 8.25
V_d_	(L/kg)	1.31 ± 0.09	1.48 ± 0.10	1.36 ± 0.11	1.49 ± 0.15	2.08 ± 0.46	1.76 ± 0.12
V_ss_	(L/kg)	1.14 ± 0.10	1.13 ± 0.03	1.00 ± 0.07	0.99 ± 0.08	1.32 ± 0.49	1.03 ± 0.10
Cl	(L/h/kg)	1.67 ± 0.19	1.60 ± 0.20	1.75 ± 0.09	1.59 ± 0.09	2.31 ± 0.48	1.90 ± 0.12
MRT_0-t_	(h)	0.61 ± 0.07	0.65 ± 0.15	0.51 ± 0.01	0.56 ± 0.05	0.55 ± 0.09	0.51 ± 0.06
MRT_0-∞_	(h)	0.69 ± 0.09	0.72 ± 0.12	0.57 ± 0.02	0.62 ± 0.03	0.56 ± 0.09	0.54 ± 0.03

**Table 6:** MS parameters for the GMDTC and metolazone (IS).

Analytes	Q1 (Da)	Q3 (Da)	DP (V)	CE (V)	CXP (V)
GMDTC	390.06	324.1	90	25	10
IS	366.0	259.2	75	30	19

## Data Availability

Not applicable.

## Supplementary Materials

The following supporting information can be downloaded at: https://www.mdpi.com/article/10.3390/molecules28031191/s1, Figure S1: Individual concentration–time curves of GMDTC in rat plasma after single intravenous infusion administration in each dose group (50, 100 and 250 mg/kg); Table S1: Concentration–time variation of GMDTC in plasma of rats (50 mg/kg) after intravenous infusion administration; Table S2: Concentration–time variation of GMDTC in plasma of rats (100 mg/kg) after intravenous infusion administration; Table S3: Concentration–time variation of GMDTC in plasma of rats (250 mg/kg) after intravenous infusion administration; Table S4: Pharmacokinetic parameters of GMDTC in plasma of rats (50 mg/kg) after intravenous infusion administration; Table S5: Pharmacokinetic parameters of GMDTC in plasma of rats (100 mg/kg) after intravenous infusion administration; Table S6: Pharmacokinetic parameters of GMDTC in plasma of rats (250 mg/kg) after intravenous infusion administration.

## Sample Availability

Samples of GMDTC are available from the authors.
