# Supplementary material for: Establishment of LC-MS/MS Method for Determination of GMDTC in Rat Plasma and Its Application in Preclinical Pharmacokinetics
Source: Molecules. 2023 Jan 25;28(3):1191. doi: 10.3390/molecules28031191 (PMC9919763; doi:10.3390/molecules28031191)
Supplement: Supplementary file 1 [file molecules-28-01191-s001.zip › molecules-2157411-supplementary.pdf]

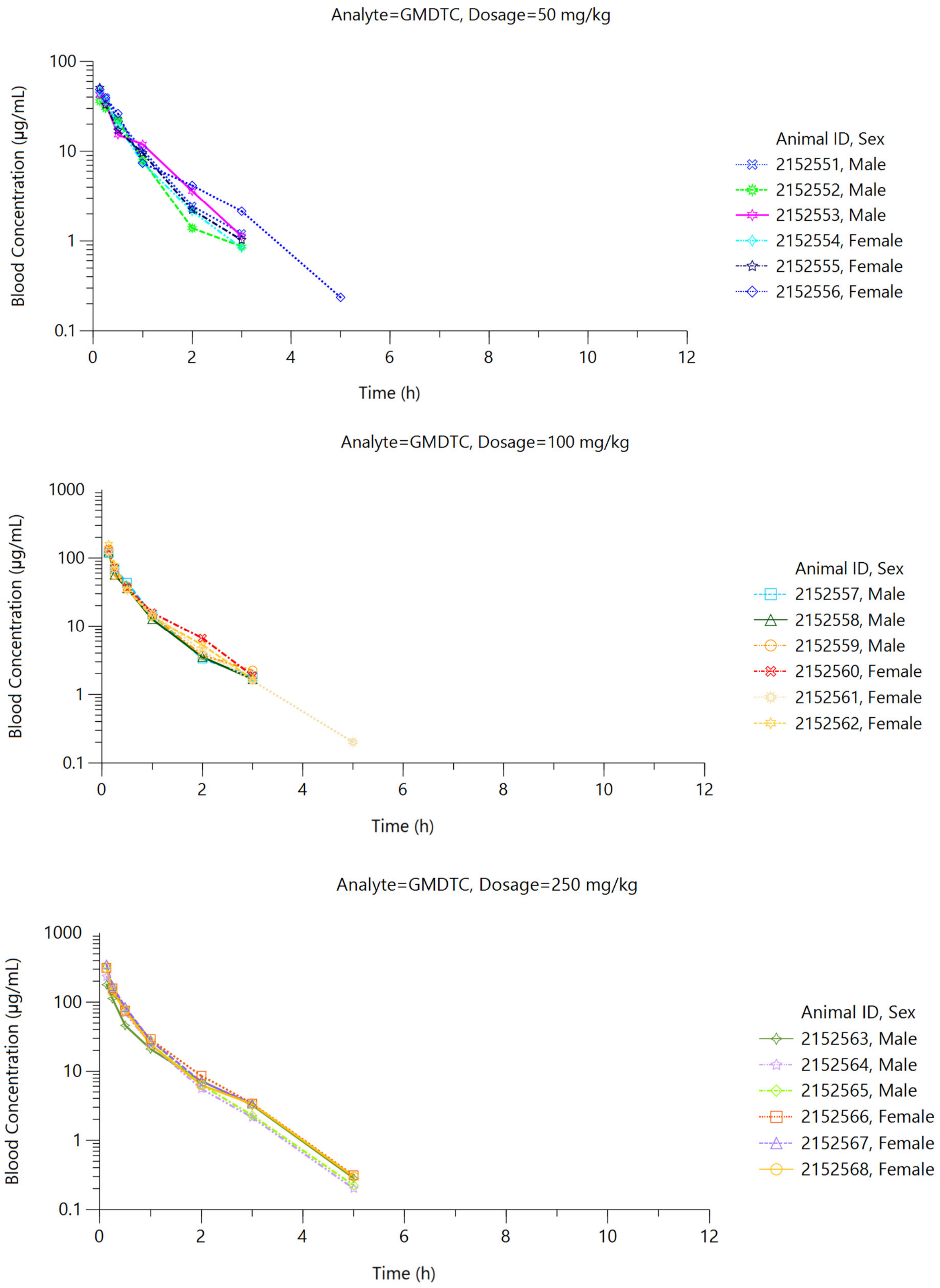

Figure S1. Individual concentration-time curves of GMDTC in rat whole blood after single intravenous infusion administration in each dose group (50, 100 and 250 mg/kg).

Table S1. Concentration-time variation of GMDTC in whole blood of rats (50 mg/kg) after intravenous infusion administration

determinand	Dose	Sex	Animal number	Time(h)									
				Before the medicine	0.133	0.25	0.5	1	2	3	5	8	12
				Whole blood concentration(μg/mL)									
GMDTC	50 mg/kg	Male	2152551	0.00	42.34	39.66	22.31	10.41	2.44	1.21	ND	ND	ND
			2152552	0.00	36.11	30.18	22.16	7.93	1.39	0.87	ND	ND	ND
			2152553	0.00	42.76	34.51	15.28	11.95	3.60	1.12	ND	ND	ND
			n	3	3	3	3	3	3	3	0	0	0
			Mean	0.00	40.40	34.78	19.92	10.10	2.48	1.07	NA	NA	NA
			RE	0.00	3.72	4.75	4.02	2.03	1.11	0.18	NA	NA	NA
			%RSD	NA	9.21	13.65	20.16	20.08	44.78	16.72	NA	NA	NA
		Female	2152554	0.00	46.48	36.30	19.60	7.46	2.13	0.83	ND	ND	ND
			2152555	0.00	51.08	32.88	17.12	9.56	2.22	1.02	ND	ND	ND
			2152556	0.00	48.79	39.41	26.30	7.38	4.17	2.14	0.24	ND	ND
			n	3	3	3	3	3	3	3	0	0	0
			Mean	0.00	48.78	36.20	21.01	8.13	2.84	1.33	0.24	NA	NA
			RE	0.00	2.30	3.26	4.75	1.24	1.15	0.71	NA	NA	NA
			%RSD	NA	4.72	9.02	22.62	15.25	40.54	53.00	NA	NA	NA

Note: 0.133 h is the immediate end of dosing.

Table S2. Concentration-time variation of GMDTC in whole blood of rats (100 mg/kg) after intravenous infusion administration

Determinand	Dose	Sex	Animal number	Time(h)									
				Before the medicine	0.133	0.25	0.5	1	2	3	5	8	12
				Whole blood concentration(μg/mL)									
GMDTC	100 mg/kg	Male	2152557	0.00	117.02	68.63	43.85	14.73	3.32	1.81	ND	ND	ND
			2152558	0.00	123.42	56.80	35.94	12.70	3.51	1.67	ND	ND	ND
			2152559	0.00	129.51	56.15	38.55	14.22	3.71	2.24	ND	ND	ND
			n	3	3	3	3	3	3	3	0	0	0
			Mean	0.00	123.32	60.53	39.45	13.89	3.52	1.91	NA	NA	NA
			RE	0.00	6.25	7.03	4.03	1.06	0.20	0.30	NA	NA	NA
			%RSD	NA	5.07	11.61	10.22	7.61	5.60	15.49	NA	NA	NA
		Female	2152560	0.00	134.99	71.89	37.04	15.83	6.64	1.88	ND	ND	ND
			2152561	0.00	121.81	68.94	34.01	15.43	4.29	1.58	0.20	ND	ND
			2152562	0.00	157.36	77.64	34.88	13.69	5.27	1.67	ND	ND	ND
			n	3	3	3	3	3	3	3	1	0	0
			Mean	0.00	138.05	72.82	35.31	14.98	5.40	1.71	0.20	NA	NA
			RE	0.00	17.98	4.43	1.56	1.14	1.18	0.15	NA	NA	NA
			%RSD	NA	13.02	6.08	4.41	7.61	21.81	8.94	NA	NA	NA

Note: 0.133 h is the immediate end of dosing.

Table S3. Concentration-time variation of GMDTC in whole blood of rats (250 mg/kg) after intravenous infusion administration

determinand	Dose	Sex	Animal number	Time(h)									
				Before the medicine	0.133	0.25	0.5	1	2	3	5	8	12
				Whole blood concentration(μg/mL)									
GMDTC	250 mg/kg	Male	2152563	0.00	179.03	112.87	46.14	21.04	7.19	3.22	0.28	ND	ND
			2152564	0.00	233.89	152.31	69.27	24.82	5.51	2.13	0.20	ND	ND
			2152565	0.00	339.39	164.17	81.33	28.34	6.30	2.29	0.22	ND	ND
			n	3	3	3	3	3	3	3	3	0	0
			Mean	0.00	250.77	143.12	65.58	24.73	6.33	2.55	0.24	NA	NA
			RE	0.00	81.50	26.86	17.89	3.65	0.84	0.59	0.04	NA	NA
			%RSD	NA	32.50	18.76	27.27	14.76	13.26	22.99	18.23	NA	NA
		Female	2152566	0.00	312.23	158.06	75.53	29.01	8.58	3.38	0.31	ND	ND
			2152567	0.00	350.53	170.12	85.21	27.95	7.04	3.39	ND	ND	ND
			2152568	0.00	310.32	140.17	75.60	24.58	6.25	3.33	0.30	ND	ND
			n	3	3	3	3	3	3	3	2	0	0
			Mean	0.00	324.36	156.12	78.78	27.18	7.29	3.37	0.30	NA	NA
			RE	0.00	22.68	15.07	5.57	2.31	1.19	0.04	0.01	NA	NA
			%RSD	NA	6.99	9.65	7.07	8.51	16.27	1.08	2.66	NA	NA

Note: 0.133 h is the immediate end of dosing.

Table S4. Pharmacokinetic parameters of GMDTC in whole blood of rats (50 mg/kg) after intravenous infusion administration

determinand	Dose	Sex	Animal number	$t_{1/2}$ (h)	T_{max} (h)	C_{max} (μg/mL)	AUC_{0-t} (h·μg/mL)	$AUC_{0-\infty}$ (h·μg/mL)	Vd (L/kg)	Vss (L/kg)	Cl (L/h/kg)	MRT_{0-t} (h)	$MRT_{0-\infty}$ (h)
GMDTC	50 mg/kg	Male	2152551	0.54	0.133	42.34	31.79	32.53	1.21	1.03	1.54	0.60	0.67
			2152552	0.51	0.133	36.11	26.13	26.57	1.37	1.14	1.88	0.55	0.60
			2152553	0.59	0.133	42.76	30.53	31.48	1.34	1.24	1.59	0.69	0.78
			n	3	3	3	3	3	3	3	3	3	3
			Mean	0.55	0.133	40.40	29.48	30.19	1.31	1.14	1.67	0.61	0.69
			RE	0.04	0.00	3.72	2.97	3.18	0.09	0.10	0.19	0.07	0.09
			%RSD	7.41	0.00	9.21	10.08	10.55	6.70	9.08	11.16	11.17	13.01
		Female	2152554	0.63	0.133	46.48	27.97	28.69	1.59	1.10	1.74	0.55	0.63
			2152555	0.60	0.133	51.08	28.74	29.51	1.46	1.13	1.69	0.58	0.67
			2152556	0.71	0.133	48.79	36.34	36.59	1.40	1.17	1.37	0.82	0.86
			n	3	3	3	3	3	3	3	3	3	3
			Mean	0.65	0.133	48.78	31.01	31.60	1.48	1.13	1.60	0.65	0.72
			RE	0.06	0.00	2.30	4.63	4.35	0.10	0.03	0.20	0.15	0.12
			%RSD	8.75	0.00	4.72	14.92	13.76	6.68	2.99	12.79	22.61	16.72

Note: 0.133 h is the immediate end of dosing.

Table S5. Pharmacokinetic parameters of GMDTC in whole blood of rats (100 mg/kg) after intravenous infusion administration

determinand	Dose	Sex	Animal number	$t_{1/2}$ (h)	T_{max} (h)	C_{max} (μg/mL)	AUC_{0-t} (h·μg/mL)	$AUC_{0-\infty}$ (h·μg/mL)	Vd (L/kg)	Vss (L/kg)	Cl (L/h/kg)	MRT_{0-t} (h)	$MRT_{0-\infty}$ (h)
GMDTC	100 mg/kg	Male	2152557	0.51	0.133	117.02	58.94	59.91	1.23	0.93	1.67	0.51	0.56
			2152558	0.54	0.133	123.42	53.21	54.21	1.43	1.04	1.84	0.51	0.57
			2152559	0.57	0.133	129.51	56.45	57.81	1.43	1.04	1.73	0.53	0.60
			n	3	3	3	3	3	3	3	3	3	3
			Mean	0.54	0.133	123.32	56.20	57.31	1.36	1.00	1.75	0.51	0.57
			RE	0.03	0.00	6.25	2.88	2.88	0.11	0.07	0.09	0.01	0.02
			%RSD	5.79	0.00	5.07	5.12	5.03	8.39	6.48	5.10	2.20	4.17
		Female	2152560	0.61	0.133	134.99	63.41	65.09	1.34	1.01	1.54	0.57	0.66
			2152561	0.68	0.133	121.81	59.07	59.27	1.65	1.06	1.69	0.61	0.63
			2152562	0.66	0.133	157.36	63.36	65.00	1.46	0.90	1.54	0.50	0.59
			n	3	3	3	3	3	3	3	3	3	3
			Mean	0.65	0.133	138.05	61.95	63.12	1.49	0.99	1.59	0.56	0.62
			RE	0.04	0.00	17.98	2.49	3.33	0.15	0.08	0.09	0.05	0.03
			%RSD	5.69	0.00	13.02	4.02	5.28	10.39	7.89	5.45	9.62	5.50

Note: 0.133 h is the immediate end of dosing.

Table S6. Pharmacokinetic parameters of GMDTC in whole blood of rats (250 mg/kg) after intravenous infusion administration

determinand	Dose	Sex	Animal number	$t_{1/2}$ (h)	T_{max} (h)	C_{max} (μg/mL)	AUC_{0-t} (h·μg/mL)	$AUC_{0-\infty}$ (h·μg/mL)	Vd (L/kg)	Vss (L/kg)	Cl (L/h/kg)	MRT_{0-t} (h)	$MRT_{0-\infty}$ (h)
GMDTC	250 mg/kg	Male	2152563	0.63	0.133	179.03	88.47	88.74	2.57	1.86	2.82	0.64	0.66
			2152564	0.62	0.133	233.89	110.69	110.88	2.02	1.18	2.25	0.51	0.52
			2152565	0.62	0.133	339.39	134.26	134.46	1.66	0.91	1.86	0.48	0.49
			n	3	3	3	3	3	3	3	3	3	3
			Mean	0.62	0.133	250.77	111.14	111.36	2.08	1.32	2.31	0.55	0.56
			RE	0.01	0.00	81.50	22.90	22.86	0.46	0.49	0.48	0.09	0.09
			%RSD	1.16	0.00	32.50	20.60	20.53	22.00	37.16	20.83	15.72	16.19
		Female	2152566	0.62	0.133	312.23	132.08	132.36	1.68	1.07	1.89	0.56	0.57
			2152567	0.66	0.133	350.53	136.68	139.57	1.70	0.92	1.79	0.44	0.51
			2152568	0.65	0.133	310.32	122.83	123.12	1.89	1.10	2.03	0.53	0.54
			n	3	3	3	3	3	3	3	3	3	3
			Mean	0.64	0.133	324.36	130.53	131.69	1.76	1.03	1.90	0.51	0.54
			RE	0.02	0.00	22.68	7.05	8.25	0.12	0.10	0.12	0.06	0.03
			%RSD	3.27	0.00	6.99	5.40	6.26	6.64	9.27	6.32	11.54	4.90

Note: 0.133 h is the immediate end of dosing.
